# Species-Specific Plant-Derived Nanoparticle Characteristics

**DOI:** 10.3390/plants11223139

**Published:** 2022-11-16

**Authors:** Akvilė Viršilė, Giedrė Samuolienė, Kristina Laužikė, Emilija Šipailaitė, Zbigniev Balion, Aistė Jekabsone

**Affiliations:** 1 Lithuanian Research Centre for Agriculture and Forestry, Institute of Horticulture, Kauno Str. 30, 54333 Babtai, Lithuania; 2 Institute of Pharmaceutical Technologies, Faculty of Pharmacy, Lithuanian University of Health Sciences, 50161 Kaunas, Lithuania

**Keywords:** antioxidant activity, plant-derived nanovesicles, medicinal plants, plant species, size distribution

## Abstract

Medicinal and agricultural plants contain numerous phytochemical compounds with pronounced biological effects on human health. They are known to encapsulate most of their characteristic bioactive compounds within membranous elements of intercellular communication known as exosomes. These nanovesicles serve as capsules protecting their biological activity and improving their penetration into the tissue. Therefore, the application of plant exosome preparations holds considerable potential for cosmetics and pharmacy, but the quality and consistency of plant material for exosome isolation is of critical importance. Therefore, in this study, we aimed to evaluate yield, size distribution patterns, and antioxidant properties between nanovesicle preparations of the following portfolio of medicinal plants: *Kalanchoe daigremontiana*, *Artemisia absinthium*, *Hypericum perforatum*, *Silybum marianum*, *Chelidonium* majus, and *Scutellaria baicalensis*. Results showed that nanoparticle yield, size distribution, and antioxidant activities were specific to plant species. Compared to other plants, nanoparticle preparations from *Artemisia absinthium* were distinguished by remarkably higher yield and concentration, while the highest antioxidant activity of plant-derived nanoparticle preparations per weight and per particle was determined to occur in *Chelidonium majus* and *Hypericum perforatum* samples. Results showed no significant correlation in DPPH (2-diphenyl-1-picrylhydrazyl) free radical scavenging activity and FRAP (ferric reducing antioxidant power) between plant material and nanoparticle preparations. More detailed biochemical analysis of exosome preparations is necessary to validate their biological activity and its relation to source plant cells.

## 1. Introduction

Extracellular vesicles are nano- and microvesicles with biogenic bilayer membranes that are secreted by various cell types in procaryotic and eukaryotic organisms and play an essential role in cellular communication by transporting bioactive cargo between cells [1]. By carrying specific proteins, nucleic acids, and other metabolites, extracellular vesicles are involved in the regulation of developmental processes, activation of the immune system, and the stress response. Moreover, they are known to mediate diverse mechanisms of intercellular, interspecies, and cross-kingdom communication [2], and these properties are important in employing extracellular vesicles as a valuable therapeutic tool [3]. The most widely investigated extracellular vesicles are exosomes, the nanovesicles of endosomal origin produced by maturation in multivesicular bodies [4]. Recently, exosome-like nanovesicles have attracted much attention as potential therapeutic agents with anti-cancer, anti-melanogenic, anti-inflammatory, anti-senescence, regenerative and other bioactivities [5,6,7,8,9]. Also, exosomes and other nanovesicles are being intensively studied as a promising drug-delivery platform [7], facilitating drug penetration into the tissues and easily passing biological barriers, including the blood-brain barrier and the placenta [6]. Such activities can be related both to the membranous structure of the nanovesicle and to its internal secondary metabolites: proteins and nucleic acids. These environment-sensitive biomolecules have been found to retain their properties only when staying encapsulated by an exosome membrane, making the vesicles unique, and therefore their bioactivity and mechanisms of action are different from plant extracts as well as from nanoparticles of other origins.

Plant-derived vesicles are recently gaining increasing attention as an alternative for mammalian biofluid- and cell culture-derived nanovesicles [5]. They have similar size distribution, surface electric charge, morphology, and density. Similar to mammalian ones, plant-derived vesicles comprise biomolecules, such as RNAs, proteins, and lipids, i.e., small metabolites that regulate physiological processes [1,3,6,10]. Moreover, plant exosomes show excellent biocompatibility, are minimally cytotoxic, do not contain zoonotic or human pathogens, are useful in reducing off-target effects, and may be derived from an abundance of plant resources for application in large-scale production [3]. Any parts of plants can serve as sources of nanovesicles for biomedical applications. The most preferred are leaves, fruits, and apoplastic fluid. It is important to note that different plant species and organs produce different amounts of nanovesicles with specific compositions and properties [7,11], therefore offering advantageous therapeutic effects stemming from the natural biochemicals from the source plant tissues [3]. Plant nanoscale particles were isolated from various plants, mainly fruits and berries (grapefruit [12], grape [13], lemon [14,15], apple [16], blueberry, orange, watermelon, pear [17], strawberry [18], and acerola [19]); vegetables (broccoli [20], soybean, tomato [17], cabbage [5,21], cucumber [22], garlic [23], onion [24], celery [25], beet [26], and carrot [27]); and some medicinal plants (ginger [28,29], ginseng [1,8], *Aloe vera* [30], *Moringa oleifera* [31], *Kaempferia parviflora* [32], *Momordica charantia* [33], *Cannabis sativa* [34], etc.). However, it is inaccurate to compare properties of nanoparticles isolated from different plant sources between studies due to uneven isolation and analysis methodologies and differential nanoparticle application objectives.

Phytochemicals with significant antioxidant activity in fruits, vegetables, and medicinal plants can be encapsulated in the lipid bilayer structure of extracellular vesicles [35], thus preserving their stability and biological activity. The membrane protects vesicle content from enzymatic degradation and other environmental influences (e.g., high and low temperatures, pH, salinity, moisture, and sunlight) [7]. Unfortunately, the available data on plant-derived vesicle antioxidant capacity and phytochemical content is very sparse and ambiguous; there was some naringenin detected in grapefruits [36], curcuminoids [37] and gingerol [38] in ginger, flavonoids in apple [16], and ascorbic acid in strawberries [18]. Nevertheless, there is barely any information about how the vesicles’ phytochemical profile and biological activity differ from those of their source material and the mechanisms by which bioactive compounds are transferred between exosomes and their parental cells.

In this study, we sought to explore the characteristics of the nanovesicles isolated from the selected portfolio of medicinal plants and known for their positive impacts on skin diseases and conditions, with potential for application in cosmetics and pharmacy [38]. However, for industrial application of nanovesicle preparations, more detailed knowledge of the specific physical and biochemical properties each plant species is necessary to ensure their high yield, purity, biological activity, safety and quality [4,10,35]. Moreover, the understanding of the relationship between antioxidant properties of exosome preparations and their source material would be beneficial for determining the quality of source plant material. Therefore, the objective of our study was to compare the yield, size, protein content, and antioxidant properties between nanovesicle preparations of different medicinal plants.

## 2. Results

**Figure 1:**
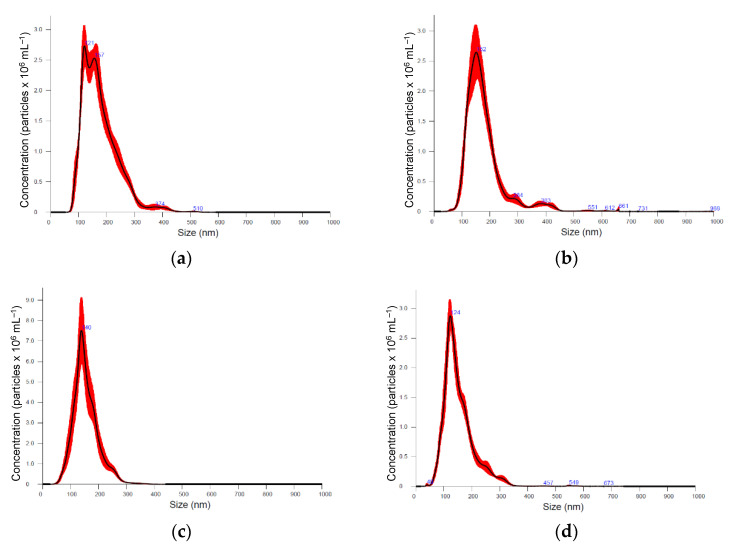

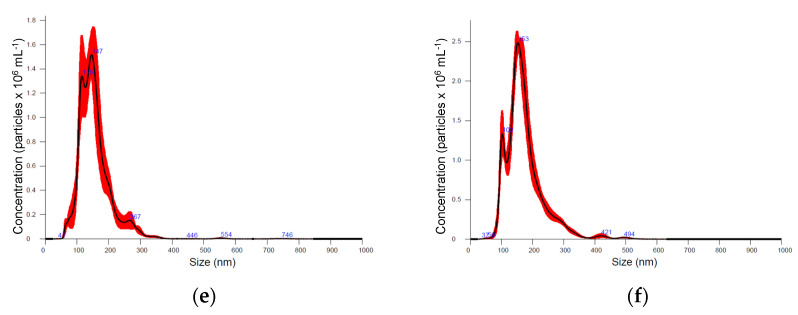
Particle size distribution according nanoparticle tracking analysis (NTA) in different medicinal plant-derived nanoparticle preparations (100× dilution): (**a**) *Kalanchoe daigremontiana*, (**b**) *Silybium marianum*, (**c**) *Artemisia absinthium*, (**d**) *Scutelaria baicalensis*, (**e**) *Chelidonium majus*, and (**f**) *Hypericum perforatum*. Black lines represent the average value (*n* = 5); the width of red band—standard deviation of the particle size distribution (*n* = 5).

Six medicinal plants, *Kalanchoe daigremontiana, Silybium marianum*, *Artemisia absinthium, Scutellaria baicalensis, Chelidonium majus* and *Hypericum perforatum* were selected for their documented biological activities and corresponding pool of secondary metabolites, the biosynthesis of which is known to be regulated by various biotic and abiotic constraints [39]. Nanoparticle preparations of comparable properties were isolated from the above-ground parts of plants, representing different plant families, morphology, and growth strategies (Figure 1, Table 1). Nanoparticle tracking analysis (NTA) results (now considered a gold standard for exosome and other nanovesicle characterisation [40]), showed no remarkable difference in mean particle size in different plants. It varied between 154 and 180 nm; *Artemisia* = *Scutelaria* ≈ *Chelidonium* < *Hypericum* ≈ *Kalanchoe* ≈ *Silybum*. The size distribution profile showed an orderly Gaussian pattern in *Silybum marianum, Artemisia absinthium*, and *Scutelaria baicalensis* (Figure 1b–d), while in other plant-derived particle preparations, size distribution was more heterogeneous. The span (Table 1), which represents the width of volume-based size distribution, according to ANOVA results, had significantly narrower values of 0.73 and 0.76 in the preparations isolated from *Artemisia absinthium* and *Chelidonium majus*, while the best recovery of size distribution between replications of measurements (the narrowest red band in Figure 1d was observed in nanoparticle isolations from *Scutelaria baicalensis*. The highest particle concentration per mg of protein, 6.75 × 10^7^, 8.43 × 10^7^, and 9.71 × 10^7^ were obtained in *Silybum marianum*, *Kalanchoe daigremontiana*, and *Artemisia absinthium* nanoparticle preparations. In the case of *Hypericum perforatum*, the high protein yield was followed by relatively low particle counts; therefore, lower particle concentration per mg of protein meant a contaminated, impure sample most likely containing co-isolated protein molecules.

Table 1 illustrates the evaluation of plant-derived nanoparticle yield characterised by protein content in the nanoparticle preparations. The yield obtained was ~1.5–2 times higher in nanoparticle isolations from the exact weight of *Artemisia absinthium* and *Hypericum perforatum* dry plant material. Particle concentration was also significantly higher in *Artemisia absinthium* preparations: 4.29 × 10^6^ particles per gram of dry plant weight, compared to 0.67–1.66 × 10^6^ particles per gram in other plant preparations. One of the explanations could be that different plant preparations varied in purity, and some of them contained more large biomolecules not encapsulated in the vesicles. It is assumed that if the prepared isolate is pure, the particle count to protein concentration ratio is relatively high and implies a low content of contaminating protein [3]. However, it is not excluded that the nanovesicles from different plants may differ in protein content and amounts, and further studies are needed to uncover which of the hypotheses is correct and to what extent.

Next to the size, count, and protein content, the antioxidant properties of the plant-derived nanoparticle isolations were characterised to predict their potential biological activity and compared with the activity in the source material. The selected plants have contrasting antioxidant properties (Table 2). *Hypericum perforatum* has ~4 times higher DPPH (2-diphenyl-1-picrylhydrazyl) free radical scavenging activity and FRAP (ferric reducing antioxidant power) compared to *Chelidonium majus*, and 1.3–1.9 times higher than that of other investigated plants. *Chelidonium majus* was also characterised by significantly lower ABTS (2,2′-azino-bis (3-ethylbenzothiazoline-6-sulphonic acid) free radical scavenging activity, which was the highest in *Artemisia absinthium* (~1.2 times higher than that of the remaining plants). In our study, the antioxidant activity of the plant material had no significant correlation with antioxidant activity of the nanoparticle preparations. DPPH free radical scavenging activity of nanoparticle preparations corresponded to 0.5–0.6% of the activity in *Kalanchoe*, *Artemisia*, and *Scutelaria*; and 1.3–1.4% of activity in *Hypericum* and *Silybium* above-ground material; while in *Chelidonium*, the transference of antioxidant DPPH free radical scavenging activity rate was relatively high, at 3.5%, compared with the activity of plant material. High DPPH and ABTS free radical activities in *Artemisia absinthium* were not transferred to its particle preparation at equal rates. FRAP antioxidant power of plant-derived nanoparticle preparations varied between 3.5–5.5% from the antioxidant power of plant material, except *Chelidonium majus* (9.7% from the FRAP antioxidant power of the plant material).

**Figure 2:**
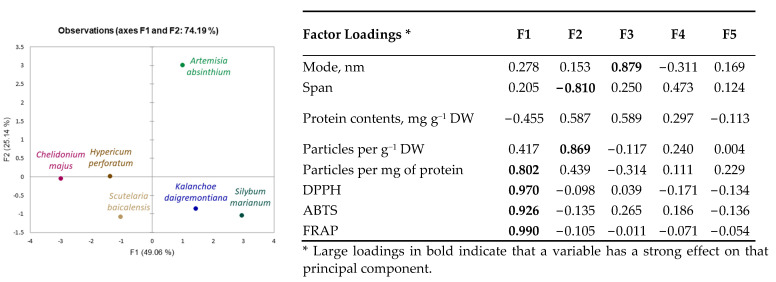
Principal component analysis (PCA) scatterplots indicating distinct differences in medicinal plant-derived nanoparticle characteristics and corresponding factor loadings.

Obtained results indicate species-specific differences in nanoparticle yield, size distribution, concentration, and antioxidant properties. A principal component analysis (PCA) scatterplot (Figure 2) confirmed distinct differences in *Artemisia absinthium* compared to other plants, and its shift on the F2 component axis, according to the factor loadings (Figure 2), was mainly related to particle concentration. Distribution of other plants was defined more by the F1 component and, according to factor loadings, primarily associated with antioxidant properties of plant-derived nanoparticle preparations. *Kalanchoe daigremontiana* and *Silybium marianum* nanoparticle preparations have similar evaluated characteristics; *Chelidonium majus* nanoparticle preparation characteristics are close to those of *Hypericum perforatum*.

## 3. Discussion

According to the obtained results, plant-derived nanovesicles bear specific properties, which can vary greatly and might be related to the biological roles of these vesicles in plants [8,41]. The first characteristic, and one that is very important for practical purposes, is particle yield. We have found it to be relatively low compared to that reported in other studies; our results indicate nanoparticle preparation represents 0.002–0.004% of the dry plant material used for isolation. Liu et al., 2020 reported 0.012, 0.028, and 0.062 mg of protein per gram of bok choi, gai lan, and cabbage, respectively [21]. Kim et al., 2021 [3] reported even higher plant-derived vesicle protein yield: 1.76 mg g^−1^ in grape, 2.21 mg g^−1^ in grapefruit, and 0.44 mg g^−1^ in tomato. However, these results cannot be directly compared due to different isolation and analysis methods. Notwithstanding, certain plants could better contribute to the large-scale production of plant exosomes, considering protein yield, interfering compounds, particle concentration, and purity. Purity was determined by the nanoparticle concentration per mg of protein. Nanoparticle preparations from *Kalanchoe daigremontiana*, *Artemisia absinthium*, and *Silybum marianum* contained 2–3 times higher counts of particles per mg of protein compared to the preparations from other studied plants, which yielded ~0.97 × 10^8^ particles per mg of protein. However, according to the literature data, isolation and purification procedures have remarkably higher impacts on the purity of nanovesicle preparations than plant species-specific differences; in studies with aloe [30] and ginger [29], after a high-purity preparation procedure, approximately 1.07 × 10^9^ and 1.3 × 10^11^ particles per mg of protein were obtained.

Dominant particle size in the nanovesicle preparations also depends on plant species, but mostly on isolation procedure, as different studies and reviews report quite ambiguous results [3,11,41]. In our study, mean particle size in different plant nanovesicle isolations varied between 154 and 180 nm with a span of 0.73–0.92. This conforms to the characteristic exosome size of 30–200 nm; to compare, other varieties of extracellular membrane structures are described as having diameters of 200–1000 nm (microvesicles) and 50–5000 nm (apoptotic bodies) [7,42]. According to this parameter, the plant-derived preparations analyzed in the current study are similar to exosomes. On the other hand, this exosome size is confirmed for human and animal exosomes, and some studies suggest that plant exosomes are generally larger than animal exosomes [43]. However, according to current operational guidelines for studying extracellular vesicles, the MISEV2018 (or minimal information for studies of extracellular vesicles 2018) [44], if the preparation is not confirmed for endosomal origin by specific positive and negative markers, it cannot be named “exosomes”. In addition, the preparation cannot be referred to as “vesicles” if the vesicular structure is not shown by electron microscopy examination; in such case, it is suggested that the preparation be titled “nanoparticles”. In our case, the isolation procedure from freeze-dried plant material could not assure all the vesicles or particles in the preparation were of extracellular origin; some might appear to be intracellular, yet derived after cell membrane rupture. Therefore, to fit the current investigation state of our preparations, we adopted the term “plant-derived nanoparticles”. Unfortunately, plant exosome-specific markers are still under research, and it is currently impossible to characterize the nanovesicle preparations according to this parameter. In addition, the comparison of extracellular vesicle size distribution in different plants and their organs (leaves, fruits, roots) between studies is aggravated by different extraction procedures employed. For example, Yamasaki et al., 2021 [24] compared extracellular vesicles from onion isolated using centrifugation at 17,000× *g* and 200,000× *g* and found that average diameters were 288.1 nm and 185.3 nm, respectively. The data obtained in our study and reported in other works suggest that a high standardisation of vesicle isolation procedures from plants is necessary to obtain recovery and homogeneity of vesicle preparations necessary for large-scale applications. According to our results, the most homogenous, uniform particle size distribution was obtained in *Scutelaria baicalensis* and *Artemisia absinthium* nanoparticle preparations using above-ground parts. Keeping in mind the relatively higher yield (0.04 mg g^−1^) and purity (0.97 × 10^8^ particles per mg of protein), *Artemisia absinthium* is one of the plants with the greatest potential for further exploration for application in cosmetics and pharmacy. However, at the same time, the biochemical properties of the nanoparticle preparations are of primary importance in employing plant exosome preparations as active cosmeceutical or therapeutical agents. Plant-derived nanovesicles are expected to contain beneficial phytochemicals from the parental cells, but the transference mechanism is largely unknown. Our results showed no significant correlation in DPPH free radical scavenging activity and FRAP antioxidant power between the plant extract and its nanoparticle preparation. Antioxidant activity of nanoparticle preparation extracts was relatively low, from 0.5 to 9.7% of the plant material, and the highest DPPH, ABTS free radical scavenging activities and FRAP antioxidant power were determined to occur in *Chelidonium majus* and *Hypericum perforatum* plant-derived nanoparticle preparations, while in source plant material extracts, DPPH free radical scavenging activity was 4 times higher and ABTS was 1.3 times higher in *Hypericum perforatum* extracts compared to that of *Chelidonium majus*. Following the idea that antioxidant compounds are encapsulated inside the particle, the antioxidant activity per single particle makes sense. DPPH free radical activity in *Chelidonium majus* and *Hypericum perforatum* nanoparticle preparations yielded ~0.4 nmol DPPH per pcs, 0.02 nmol DPPH per pcs in *Artemisia absinthium*, and 0.1–0.2 nmol DPPH per pcs in other investigated plant preparations. Similar trends were obtained with ABTS free radical activity and FRAP antioxidant power results. The results suggest that the number, size, protein content characteristics, and antioxidant properties of plant-derived nanoparticle preparations, which define their target biological activity, cannot be rated directly and unambiguously, especially when the mechanisms of vesicle “packaging” with antioxidant compounds are unclear. Woith et al., 2021 [35] analyzed a list of plant extracellular vesicle samples and found that lipophilic compounds were associated with nanovesicles, while more hydrophilic structures were not consistently found, therefore concluding that secondary metabolites might not be actively packaged into extracellular vesicles, but are enriched in the membrane when they are lipophilic enough. Shkryl et al., 2022 [7] presumed that small secondary metabolite molecules are encapsulated into the exosomes unspecifically, via passive diffusion. Moreover, the biological activity of exosome preparation is not defined only by antioxidant compounds but by the complex of protein, nucleic acids, sugars, and other primary and secondary metabolites. Therefore, a more detailed biochemical analysis of exosome preparations is necessary to validate their biological activity.

## 4. Materials and Methods

Object and cultivation conditions. *Kalanchoe daigremontiana* (Crassulaceae), with the common name mother of thousands; *Silybium marianum* (Asteraceae), with the common name milk thistle; *Artemisia absinthium* (Asteraceae), with the common name wormwood, *Scutellaria baicalensis* (Lamiaceae), with the common name Baikal skullcap, *Chelidonium majus* (Papaveraceae), with the common name greater celandine; and *Hypericum perforatum* (Hypericaceae), with the common name St. John’s wort were cultivated in controlled-environment chambers under constant environmental conditions with the aim of maintaining equal plant production quality despite the cultivation season. The following conditions were maintained: 21/17 °C day/night temperature, relative air humidity ~55%, 16-h photo/thermo period, and LED (Tungsram Agritech Research Toplight research module, Budapest, Hungary) lighting photosynthetic photon flux density ~250 µmol m^−2^s^−1^ at the top of the plant. The lighting spectrum consisted of deep red 61%, blue 20%, white 15%, and far-red 4%. Plants were cultivated from seeds, except *Kalanchoe daigremontiana*, which was cultivated from plantlets in peat substrate (Profi 1, Durpeta, Šepeta, Lithuania) in 450 mL-volume plastic containers, watered when needed, and fertilized with liquid NPK 3-1-3 fertilizer with microelements (Palgron, Ospel, The Netherlands). Plants were cultivated under constant conditions for 30–60 days from sowing until the species-specific butonisation (beginning of flowering) stage.

Nanoparticle isolation. At the end of the cultivation period, above-ground plant material was collected, frozen in liquid nitrogen, lyophilised (FD-7, SIA Cryogenic and Vacuum Systems, Ventspils, Latvia), and ground. A nanoparticle isolation procedure was performed with the dry plant material using an isolation kit (2-EPL, Exolitus, Kaunas, Lithuania) based on stabilisation, precipitation and purification of exosomes using low-speed centrifugation (Z366, Hermle, Gosheim, Germany). Nanoparticle preparations isolated from 1 g of dry plant material were resuspended in 0.5 mL of PBS buffer for size distribution analysis and protein content analysis, and in 0.5 mL of 80% methanol for antioxidant activity evaluation.

Nanoparticle tracking analysis (NTA) was employed to confirm the size distribution and concentration of the nanovesicles using a NanoSight NS300 system (Malvern Technologies, Malvern, UK). The prepared samples were diluted 100-fold with PBS and were measured using NTA five times to obtain the average of the mean particle size, the span of particle size distribution, and particle concentration in nanoparticle isolate suspension. Span = (D90−D10)/D50, where D10, D50, and D90 signify the points in the size distribution, up to and including those at which 10, 50, and 90% of the total volume of particles in the sample was contained. Obtained particle concentrations were re-calculated to represent the number of particles per 1 g of plant dry weight (DW) and per 1 mg of plant-derived nanoparticle protein.

Protein contents were evaluated in nanoparticle isolates and resuspended in PBS by the Bradford method according to the calibration curve of bovine serum albumin (0.05–1.0 mg mL^−1^). Volumes of 10 μL of sample/standard were mixed with 190 μL of Bradford reagent and absorption was measured at 595 nm (Spectro-star Nano, BMG Labtech microplate reader, Ortenberg, Germany). Final plant-derived nanoparticle protein contents were expressed as mg of protein per 1 g of plant dry weight (DW).

Antioxidant activity was evaluated in plant material and nanoparticle extracts. Plant extracts were prepared by grinding 0.01 g of dry plant material with 5 mL of 80% methanol, incubated for 24 h, and centrifuged (4500 rpm; Z366, Hermle, Gosheim, Germany). Plant-derived nanoparticle isolates, resuspended in 80% methanol, were used directly for antioxidant analysis. Each measurement was performed in 3 replications. Antioxidant properties were evaluated as: DPPH, ABTS free radical scavenging activity, and ferric reduction antioxidant power (FRAP).

For DPPH (2-diphenyl-1-picrylhydrazyl) assay, a stable 126.8 μM DPPH (100% pu-rity, Sigma-Aldrich, Burlington, MA, USA) solution was prepared in methanol [45]. A volume of 290 μL of the DPPH solution was transferred to a test tube and mixed with 20 μL of the plant-derived nanoparticle extract. The absorbance was read at 515 nm (Spectro-star Nano, BMG Labtech microplate reader, Ortenberg, Germany) at 16^th^-min intervals. DPPH free radical scavenging activity was expressed as mmol of DPPH per 1 g of dry plant weight (µmol g^–1^ DW) or per plant-derived nanoparticle preparation, isolated from 1 g of dry plant weight.

The ABTS (2,2′-azino-bis (3-ethylbenzothiazoline-6-sulphonic acid)) radical cation was obtained by incubating the 7 mM ABTS stock solution with 2.45 mM potassium persulfate K_2_S_2_O_8_ for 12–16 h in the dark before use [46]. Thereafter, 20 μL of the prepared sample was mixed with 290 μL of diluted (1:7) ABTS solution and the absorbance was measured after 11 min (plateau phase) at 734 nm (Spectrostar Nano, BMG Labtech microplate reader, Ortenberg Germany). The ABTS scavenging activity of medicinal plant material and plant-derived nanoparticle extracts was calculated as the difference between the initial absorbance and after reacting for 11 min, and the final result was expressed as mmol ABTS scavenged by 1 g of dry plant weight (µmol g^–1^ DW) or by plant-derived nanoparticle preparation, isolated from 1 g of dry plant weight.

The FRAP (ferric reducing antioxidant power) method is based on plant extract antioxidant power to reduce ferric ion (Fe^3+^) to ferrous ions (Fe^2+^). The fresh working solution was prepared by mixing 300 mM of pH 3.6 acetate buffer, 10 mM TPTZ (2,4,6-tripyridyl-s-triazine) solution in 40 mM HCl, and 20 mM FeCl_3_ × 6H_2_O at 10:1:1 (*v/v/v*) [47]. A volume of 20 µL of the sample was mixed with 290 μL of working solution and incubated in the dark for 30 min. Then, absorbance was read at 593 nm (Spectrostar Nano BMG Labtech microplate reader, Germany). A calibration curve was determined using 0.005–0.5 mM Fe_2_(SO_4_)_3_ (Iron (III) sulphate (97% purity, Sigma-Aldrich, Burlington, MA, USA). The antioxidant power was expressed as µmol of Fe^2+^ reduced by g^−1^ of dry plant weight (DW, or by plant-derived nanoparticle preparation, isolated from 1 g of plant DW).

Statistical analysis. Nanoparticles were isolated from plant material in 3 replications. The results are presented as the average of 5 (NTA analysis) or 3 (antioxidant activity, protein content) ± standard deviation (SD). For result modelling, ANOVA analysis using Tukey’s test at the confidence level *p* ≤ 0.05, correlation analysis, and multivariate principal component analysis (PCA) were performed. Data were evaluated using MS Excel and compatible XLStat 2021.5 (Addinsoft, Paris, France) software packages.

## 5. Conclusions

Plant-derived nanovesicle preparations can be obtained from the abundance of plant resources; however, vesicle characteristics must be evaluated individually for their safety and efficiency because their numbers, size distribution, and antioxidant activity vary significantly between plant species and will be affected by the phytochemical properties of the source plant material. Compared to other investigated plants, nanoparticle preparations from *Artemisia absinthium* were distinguished by remarkably higher yield and concentration, while the highest antioxidant activity of plant-derived nanoparticle preparations per weight and per particle was determined to occur in *Chelidonium majus* and *Hypericum perforatum* samples. Nanovesicle yield, particle concentration, and antioxidant content should be assessed together. Results expressed through particle concentration provide more accurate insights when compared to direct parameters. Therefore, intensive studies of plant extracellular vesicle biogenesis and composition are urgently required to define positive and negative biomarkers for plant exosomes, ectovesicles, and other extracellular vesicle subclasses.

## Figures and Tables

**Table 1:** Plant-derived nanoparticle yield, size, and number distribution ($\bar{x}\pm SD,\;n=5)$. Different letters within the column indicate statistically significant differences between means according to Tukey’s test at the confidence level *p* ≤ 0.05.

Medicinal Plant	Protein Content, mg g^−1^ DW *	Particle Size, nm		Particle Concentration	
		Mean	Span **	Pcs Per g^−1^ of DW	Pcs Per mg^−1^ of Protein
*Kalanchoe daigremontiana*	0.02 ± 0.009 ^C^	177 ± 3.8 ^A^	0.91 ^A^	1.66 ± 0.12 × 10^6 B^	8.43 × 10^7 A^
*Silybum marianum*	0.02 ± 0.015 ^C^	180 ± 2.2 ^A^	0.87 ^A^	1.35 ± 0.11 × 10^6 B^	6.75 × 10^7 A^
*Artemisia absinthium*	0.04 ± 0.013 ^A^	154 ± 1.9 ^B^	0.73 ^B^	4.29 ± 0.83 × 10^6 A^	9.71 × 10^7 B^
*Scutellaria baicalensis*	0.03 ± 0.011 ^B^	154 ± 2.4 ^B^	0.92 ^A^	1.21 ± 0.05 × 10^6 B^	3.55 × 10^7 B^
*Chelidonium majus*	0.03 ± 0.015 ^B^	156 ± 2.6 ^B^	0.76 ^B^	0.67 ± 0.01 × 10^6 B^	2.25 × 10^7 A^
*Hypericum perforatum*	0.05 ± 0.017 ^A^	174 ± 1.3 ^A^	0.88 ^A^	1.14 ± 0.09 × 10^6 B^	2.24 × 10^7 B^

* Protein amount, equivalent to nanoparticle yield from 1 g of dry plant weight (DW). ** Span = (D90 − D10)/D50, where D10, D50, and D90 signify the point in the size distribution, up to and including that for which 10, 50, and 90% of the total volume of particles in the sample are contained.

**Table 2:** Antioxidant properties of plant material and plant-derived nanoparticle preparations ($\bar{x}\pm SD,\;n=3)$. Different letters within the column indicate statistically significant differences between means, according to Tukey’s test, at the confidence level *p* ≤ 0.05. Determination coefficient represents the corelation between measured antioxidant parameters in plant extract and nanoparticle preparation.

Medicinal Plant	DPPH Scavenging Activity, mmol g^−1^ DW		ABTS Scavenging Activity, µmol g^−1^ DW		FRAP, µmol Fe(II) g^−1^ DW	
	Plant Material	Nanoparticle Preparation	Plant Material	Nanoparticle Preparation	Plant Material	Nanoparticle Preparation
*Kalanchoe daigremontiana*	27.59 ± 0.98 ^B^	0.16 ± 0.04 ^C^	127.98 ± 0.52 ^B^	1.01 ± 0.21 ^B^	10.51 ± 1.12 ^CD^	0.47 ± 0.01 ^CD^
*Silybum marianum*	17.18 ± 0.47 ^C^	0.26 ± 0.02 ^B^	122.05 ± 0.52 ^C^	1.10 ± 0.11 ^B^	9.47 ± 0.33 ^D^	0.53 ± 0.02 ^BC^
*Artemisia absinthium*	17.23 ± 1.08 ^C^	0.08 ± 0.03 ^D^	157.50 ± 1.90 ^A^	0.38 ± 0.17 ^C^	12.25 ± 0.06 ^BC^	0.40 ± 0.00 ^E^
*Scutellaria baicalensis*	25.78 ± 0.47 ^B^	0.15 ± 0.03 ^CD^	128.42 ± 0.22 ^B^	0.99 ± 0.01 ^B^	12.93 ± 0.78 ^B^	0.45 ± 0.01 ^DE^
*Chelidonium majus*	8.03 ± 0.62 ^D^	0.28 ± 0.02 ^B^	78.96 ± 0.07 ^D^	1.17 ± 0.03 ^AB^	5.76 ± 0.95 ^E^	0.56 ± 0.41 ^B^
*Hypericum perforatum*	33.60 ± 0.10 ^A^	0.45 ± 0.01 ^A^	127.10 ± 0.44 ^B^	1.55 ± 0.09 ^A^	18.13 ± 0.41 ^A^	0.64 ± 0.02 ^A^
R^2^ (Plant × Nanoparticle)	0.283		–0.520		0.216	

## Data Availability

Not applicable.

## References

[1] Yang S., Lu S., Ren L., Bian S., Zhao D., Liu M., Wang J. Ginseng-Derived Nanoparticles Induce Skin Cell Proliferation and Promote Wound Healing. J. Ginseng Res..

[2] Urzì O., Raimondo S., Alessandro R. (2021). Extracellular Vesicles from Plants: Current Knowledge and Open Questions. Int. J. Mol. Sci..

[3] Kim J., Li S., Zhang S., Wang J. (2022). Plant-Derived Exosome-like Nanoparticles and Their Therapeutic Activities. Asian J. Pharm. Sci..

[4] Zhang Y., Liu Y., Liu H., Tang W.H. (2019). Exosomes: Biogenesis, Biologic Function and Clinical Potential. Cell Biosci..

[5] You J.Y., Kang S.J., Rhee W.J. (2021). Isolation of Cabbage Exosome-like Nanovesicles and Investigation of Their Biological Activities in Human Cells. Bioact. Mater..

[6] Logozzi M., Di Raimo R., Mizzoni D., Fais S. (2022). The Potentiality of Plant-Derived Nanovesicles in Human Health—A Comparison with Human Exosomes and Artificial Nanoparticles. Int. J. Mol. Sci..

[7] Shkryl Y., Tsydeneshieva Z., Degtyarenko A., Yugay Y., Balabanova L., Rusapetova T., Bulgakov V. (2022). Plant Exosomal Vesicles: Perspective Information Nanocarriers in Biomedicine. Appl. Sci..

[8] Cho E.-G., Choi S.-Y., Kim H., Choi E.-J., Lee E.-J., Park P.-J., Ko J., Kim K.P., Baek H.S. (2021). Panax Ginseng-Derived Extracellular Vesicles Facilitate Anti-Senescence Effects in Human Skin Cells: An Eco-Friendly and Sustainable Way to Use Ginseng Substances. Cells.

[9] Cho J.H., Hong Y.D., Kim D., Park S.J., Kim J.S., Kim H.-M., Yoon E.J., Cho J.-S. (2022). Confirmation of Plant-Derived Exosomes as Bioactive Substances for Skin Application through Comparative Analysis of Keratinocyte Transcriptome. Appl. Biol. Chem..

[10] Shinge S.A.U., Xiao Y., Xia J., Liang Y., Duan L. (2022). New Insights of Engineering Plant Exosome-like Nanovesicles as a Nanoplatform for Therapeutics and Drug Delivery. Extracell. Vesicles Circ. Nucleic Acids.

[11] Nemati M., Singh B., Mir R.A., Nemati M., Babaei A., Ahmadi M., Rasmi Y., Golezani A.G., Rezaie J. (2022). Plant-Derived Extracellular Vesicles: A Novel Nanomedicine Approach with Advantages and Challenges. Cell Commun. Signal..

[12] Wang Q., Ren Y., Mu J., Egilmez N.K., Zhuang X., Deng Z., Zhang L., Yan J., Miller D., Zhang H.-G. (2015). Grapefruit-Derived Nanovectors Use an Activated Leukocyte Trafficking Pathway to Deliver Therapeutic Agents to Inflammatory Tumor Sites. Cancer Res..

[13] Ju S., Mu J., Dokland T., Zhuang X., Wang Q., Jiang H., Xiang X., Deng Z.-B., Wang B., Zhang L. (2013). Grape Exosome-like Nanoparticles Induce Intestinal Stem Cells and Protect Mice From DSS-Induced Colitis. Mol. Ther..

[14] Takakura H., Nakao T., Narita T., Horinaka M., Nakao-Ise Y., Yamamoto T., Iizumi Y., Watanabe M., Sowa Y., Oda K. (2022). Citrus Limon L.-Derived Nanovesicles Show an Inhibitory Effect on Cell Growth in P53-Inactivated Colorectal Cancer Cells via the Macropinocytosis Pathway. Biomedicines.

[15] Raimondo S., Naselli F., Fontana S., Monteleone F., Lo Dico A., Saieva L., Zito G., Flugy A., Manno M., Di Bella M.A. (2015). *Citrus Limon* -Derived Nanovesicles Inhibit Cancer Cell Proliferation and Suppress CML Xenograft Growth by Inducing TRAIL-Mediated Cell Death. Oncotarget.

[16] Fujita D., Arai T., Komori H., Shirasaki Y., Wakayama T., Nakanishi T., Tamai I. (2018). Apple-Derived Nanoparticles Modulate Expression of Organic-Anion-Transporting Polypeptide (OATP) 2B1 in Caco-2 Cells. Mol. Pharm..

[17] Xiao J., Feng S., Wang X., Long K., Luo Y., Wang Y., Ma J., Tang Q., Jin L., Li X. (2018). Identification of Exosome-like Nanoparticle-Derived MicroRNAs from 11 Edible Fruits and Vegetables. PeerJ.

[18] Perut F., Roncuzzi L., Avnet S., Massa A., Zini N., Sabbadini S., Giampieri F., Mezzetti B., Baldini N. (2021). Strawberry-Derived Exosome-Like Nanoparticles Prevent Oxidative Stress in Human Mesenchymal Stromal Cells. Biomolecules.

[19] Umezu T., Takanashi M., Murakami Y., Ohno S., Kanekura K., Sudo K., Nagamine K., Takeuchi S., Ochiya T., Kuroda M. (2021). Acerola Exosome-like Nanovesicles to Systemically Deliver Nucleic Acid Medicine via Oral Administration. Mol. Ther.–Methods Clin. Dev..

[20] Deng Z., Rong Y., Teng Y., Mu J., Zhuang X., Tseng M., Samykutty A., Zhang L., Yan J., Miller D. (2017). Broccoli-Derived Nanoparticle Inhibits Mouse Colitis by Activating Dendritic Cell AMP-Activated Protein Kinase. Mol. Ther..

[21] Liu Y., Wu S., Koo Y., Yang A., Dai Y., Khant H., Osman S.R., Chowdhury M., Wei H., Li Y. (2020). Characterisation of and Isolation Methods for Plant Leaf Nanovesicles and Small Extracellular Vesicles. Nanomed. Nanotechnol. Biol. Med..

[22] Abraham A.M., Wiemann S., Ambreen G., Zhou J., Engelhardt K., Brüßler J., Bakowsky U., Li S.-M., Mandic R., Pocsfalvi G. (2022). Cucumber-Derived Exosome-like Vesicles and PlantCrystals for Improved Dermal Drug Delivery. Pharmaceutics.

[23] Özkan İ., Koçak P., Yıldırım M., Ünsal N., Yılmaz H., Telci D., Şahin F. (2021). Garlic (*Allium sativum*)-Derived SEVs Inhibit Cancer Cell Proliferation and Induce Caspase Mediated Apoptosis. Sci. Rep..

[24] Yamasaki M., Yamasaki Y., Furusho R., Kimura H., Kamei I., Sonoda H., Ikeda M., Oshima T., Ogawa K., Nishiyama K. (2021). Onion (*Allium Cepa* L.)-Derived Nanoparticles Inhibited LPS-Induced Nitrate Production, However, Their Intracellular Incorporation by Endocytosis Was Not Involved in This Effect on RAW264 Cells. Molecules.

[25] Taşlı P.N. (2022). Usage of Celery Root Exosome as an Immune Suppressant; Lipidomic Characterization of *Apium graveolens* Originated Exosomes and Its Suppressive Effect on PMA /Ionomycin Mediated CD4^+^ T Lymphocyte Activation. J. Food Biochem..

[26] Mahdipour E. (2022). *Beta Vulgaris* Juice Contains Biologically Active Exosome-like Nanoparticles. Tissue Cell.

[27] Kim D.K., Rhee W.J. (2021). Antioxidative Effects of Carrot-Derived Nanovesicles in Cardiomyoblast and Neuroblastoma Cells. Pharmaceutics.

[28] Zhang M., Wang X., Han M.K., Collins J.F., Merlin D. (2017). Oral Administration of Ginger-Derived Nanolipids Loaded with SiRNA as a Novel Approach for Efficient SiRNA Drug Delivery to Treat Ulcerative Colitis. Nanomedicine.

[29] Teng Y., Ren Y., Sayed M., Hu X., Lei C., Kumar A., Hutchins E., Mu J., Deng Z., Luo C. (2018). Plant-Derived Exosomal MicroRNAs Shape the Gut Microbiota. Cell Host Microbe.

[30] Kim M.K., Choi Y.C., Cho S.H., Choi J.S., Cho Y.W. (2021). The Antioxidant Effect of Small Extracellular Vesicles Derived from Aloe Vera Peels for Wound Healing. Tissue Eng. Regen. Med..

[31] Potestà M., Roglia V., Fanelli M., Pietrobono E., Gismondi A., Vumbaca S., Nguedia Tsangueu R.G., Canini A., Colizzi V., Grelli S. (2020). Effect of Microvesicles from Moringa Oleifera Containing MiRNA on Proliferation and Apoptosis in Tumor Cell Lines. Cell Death Discov..

[32] Nemidkanam V., Chaichanawongsaroj N. (2022). Characterizing Kaempferia Parviflora Extracellular Vesicles, a Nanomedicine Candidate. PLoS ONE.

[33] Cai H., Huang L.-Y., Hong R., Song J.-X., Guo X.-J., Zhou W., Hu Z.-L., Wang W., Wang Y.-L., Shen J.-G. (2022). Momordica Charantia Exosome-Like Nanoparticles Exert Neuroprotective Effects Against Ischemic Brain Injury via Inhibiting Matrix Metalloproteinase 9 and Activating the AKT/GSK3β Signaling Pathway. Front. Pharmacol..

[34] Tajik T., Baghaei K., Moghadam V.E., Farrokhi N., Salami S.A. (2022). Extracellular Vesicles of Cannabis with High CBD Content Induce Anticancer Signaling in Human Hepatocellular Carcinoma. Biomed. Pharmacother..

[35] Woith E., Guerriero G., Hausman J.-F., Renaut J., Leclercq C.C., Weise C., Legay S., Weng A., Melzig M.F. (2021). Plant Extracellular Vesicles and Nanovesicles: Focus on Secondary Metabolites, Proteins and Lipids with Perspectives on Their Potential and Sources. Int. J. Mol. Sci..

[36] Wang B., Zhuang X., Deng Z.-B., Jiang H., Mu J., Wang Q., Xiang X., Guo H., Zhang L., Dryden G. (2014). Targeted Drug Delivery to Intestinal Macrophages by Bioactive Nanovesicles Released from Grapefruit. Mol. Ther..

[37] Zhuang X., Deng Z.-B., Mu J., Zhang L., Yan J., Miller D., Feng W., McClain C.J., Zhang H.-G. (2015). Ginger-Derived Nanoparticles Protect against Alcohol-Induced Liver Damage. J. Extracell. Vesicles.

[38] Wang Y., Wang J., Ma J., Zhou Y., Lu R. (2022). Focusing on Future Applications and Current Challenges of Plant Derived Extracellular Vesicles. Pharmaceuticals.

[39] Khare S., Singh N.B., Singh A., Hussain I., Niharika K., Yadav V., Bano C., Yadav R.K., Amist N. (2020). Plant Secondary Metabolites Synthesis and Their Regulations under Biotic and Abiotic Constraints. J. Plant Biol..

[40] Comfort N., Cai K., Bloomquist T.R., Strait M.D., Ferrante A.W., Baccarelli A.A. (2021). Nanoparticle Tracking Analysis for the Quantification and Size Determination of Extracellular Vesicles. J. Vis. Exp..

[41] Alfieri M., Leone A., Ambrosone A. (2021). Plant-Derived Nano and Microvesicles for Human Health and Therapeutic Potential in Nanomedicine. Pharmaceutics.

[42] Narauskaitė D., Vydmantaitė G., Rusteikaitė J., Sampath R., Rudaitytė A., Stašytė G., Aparicio Calvente M.I., Jekabsone A. (2021). Extracellular Vesicles in Skin Wound Healing. Pharmaceuticals.

[43] Karamanidou T., Tsouknidas A. (2022). Plant-Derived Extracellular Vesicles as Therapeutic Nanocarriers. Int. J. Mol. Sci..

[44] Théry C., Witwer K.W., Aikawa E., Alcaraz M.J., Anderson J.D., Andriantsitohaina R., Antoniou A., Arab T., Archer F., Atkin-Smith G.K. (2018). Minimal Information for Studies of Extracellular Vesicles 2018 (MISEV2018): A Position Statement of the International Society for Extracellular Vesicles and Update of the MISEV2014 Guidelines. J. Extracell. Vesicles.

[45] Sharma O.P., Bhat T.K. (2009). DPPH Antioxidant Assay Revisited. Food Chem..

[46] Re R., Pellegrini N., Proteggente A., Pannala A., Yang M., Rice-Evans C. (1999). Antioxidant Activity Applying an Improved ABTS Radical Cation Decolorization Assay. Free Radic. Biol. Med..

[47] Benzie I.F.F., Strain J.J. (1996). The Ferric Reducing Ability of Plasma (FRAP) as a Measure of “Antioxidant Power”: The FRAP Assay. Anal. Biochem..

